# Jejunal transcriptomic profiling of two layer strains throughout the entire production period

**DOI:** 10.1038/s41598-021-99566-5

**Published:** 2021-10-11

**Authors:** Adewunmi Omolade Omotoso, Henry Reyer, Michael Oster, Siriluck Ponsuksili, Nares Trakooljul, Eduard Muráni, Vera Sommerfeld, Markus Rodehutscord, Klaus Wimmers

**Affiliations:** 1 Institute for Genome Biology, Research Institute for Farm Animal Biology (FBN), Wilhelm-Stahl-Allee 2, 18196 Dummerstorf, Germany; 2 Institute of Animal Science, University of Hohenheim, Emil-Wolff-Str. 10, 70599 Stuttgart, Germany; 3 Faculty of Agricultural and Environmental Sciences, University Rostock, Justus-von-Liebig-Weg 7, 18059 Rostock, Germany

**Keywords:** Animal breeding, Development, Functional genomics, Gene expression, Transcriptomics, Hormones

## Abstract

The jejunum plays crucial roles for the digestion and absorption of nutrients and minerals and for barrier functions that are essential for a healthy, productive life cycle of farm animals, including laying hens. Accordingly, knowledge of the molecular pathways that emerge in the intestine during development, and particularly at the beginning of laying activity, will help to derive strategies for improving nutrient efficiency in laying hens. In this study, jejunal samples were obtained from two high-yielding layer strains at five developmental stages (weeks 10, 16, 24, 30 and 60 of life) for RNA-sequencing, alongside the profiling of blood plasma parameters to approximate the dynamics of mineral homeostasis. The results reflected a marked distinction between the pre-laying and laying phase as inferred from levels of parathyroid hormone, triiodothyronine, estradiol, vitamin D, and calcium. Moreover, the expression patterns of the intestinal mucosa responded directly to the changing metabolic and nutritional profiles at the beginning of the laying phase in maturing high-yielding strains of laying hens. These comprise signaling events namely RANK/RANKL signaling and cellular senescence. Taken together, the timing of sexual maturity of laying hens demands closer examination to unravel metabolic requirements and associated endogenous mechanisms.

## Introduction

Laying hens provide an affordable, safe and high-quality animal protein source in the form of eggs required to meet the nutritional demands of the growing human population which currently stands at 7.7 billion^1^. The global annual egg production is estimated at 76 million tonnes^2,3^, showing a trend of the continuous increase in the number of layers, achieved mainly by populous countries such as China and India in an attempt to mitigate food insecurity associated challenges^4^. The European Union accounts for the second-largest share of world egg production after China, with an estimated number of over 400 million laying hens. Animals of the Lohmann Brown (LB) and Lohmann Selected Leghorn (LSL) strains are widely used across husbandry systems^5,6^. Both LB and LSL strains have been improved for egg production performance and have been extensively monitored at the levels of bone quality, egg quality and behaviour^7^. Their egg production performance is approximately identical, however, LB and LSL layer strains significantly differ in gene expression profiles of cerebrum, egg quality parameters (egg and eggshell weights), mineral metabolism (bone-breaking strength, phytate degradation, trans- and paracellular transport), and immune responsiveness^8–12^. Importantly, the attainment of sexual maturity in pullets (~ 18 weeks) through to the onset of laying (~ 24 weeks) represents a significant physiological shift within the layers’ metabolic demand. More so, this developmental phase encompasses the cumulative inputs and interconnectivity of different biological factors spanning nutrients and mineral metabolism, neuro-endocrinal complexes, hepatic, skeletal and the immune systems^13–15^. The small intestine, specifically the jejunum is tasked with the vital role of nutrient and mineral absorption (e.g., glucose, calcium, phosphorus), amongst other regulatory and crucial functions such as, barrier integrity, immune defense, lipid metabolism and endocrinal functions all of which ultimately contribute to the overall health and stability in production performance of the hens^16,17^.

Furthermore, the developmental transition of the layers from pullets to growers (pre-layers) to layers (onset of lay), its peak in egg production and the senescence are strongly mediated intrinsically by the temporal expression of gene transcripts^18,19^, supported by the dynamics of the endocrine status and their interaction with the environment to depict these physiological outcomes. Thus, the dietary regimen for layers is adjusted to meet requirements for the respective production stages, e.g. higher dietary calcium at the onset of laying (3.5–4.5% in dry matter) compared to grower phase (0.9–1.2% in dry matter) and pre-layer phase (2.0–2.5% in dry matter)^20^, albeit this recommendation might be outdated and in need of a scientific re-evaluation^9,21^.

Transcriptomics, a current genomic appraisal method widely employed in the study of several species populations, laying hens inclusive, provides relative ease in the detection of differential gene expression. Thus, its use as a genomic appraisal tool is quite significant. Transcriptomic studies have been conducted with the Lohmann layers to uncover temporal differential gene expression patterns in oviduct development and defense in pre-laying and laying hens^22^. Conversely, transcriptomic insight into the developmental process in laying hens through the enteral routes (jejunum) continues to be limited. Clearly, intrinsic mechanisms throughout the entire production period including the utilization of nutrients should be exploited. The multifaceted function and synergistic inclusion of the small intestine in various biological complexes are associated with development and maturity in the laying hens, coupled with the similarities in production and the different adaptive strategies adopted for mineral homeostasis, immune and bone traits. We hypothesize that knowledge of differentially expressed genes (DEGs) and molecular pathways related to development, growth and the onset of laying will contribute to further improvements in nutrient efficiency and productivity.

The present study investigated differentially abundant mRNA transcripts and enriched pathways in the jejunum of two-layer strains (LB and LSL). Jejunal samples were collected throughout the entire production period at weeks 10, 16, 24, 30 and 60 of life for high-throughput RNA sequencing, incorporating blood parameters to approximate the dynamics of mineral homeostasis.

## Results

### Blood plasma profiling

**Figure 1:**
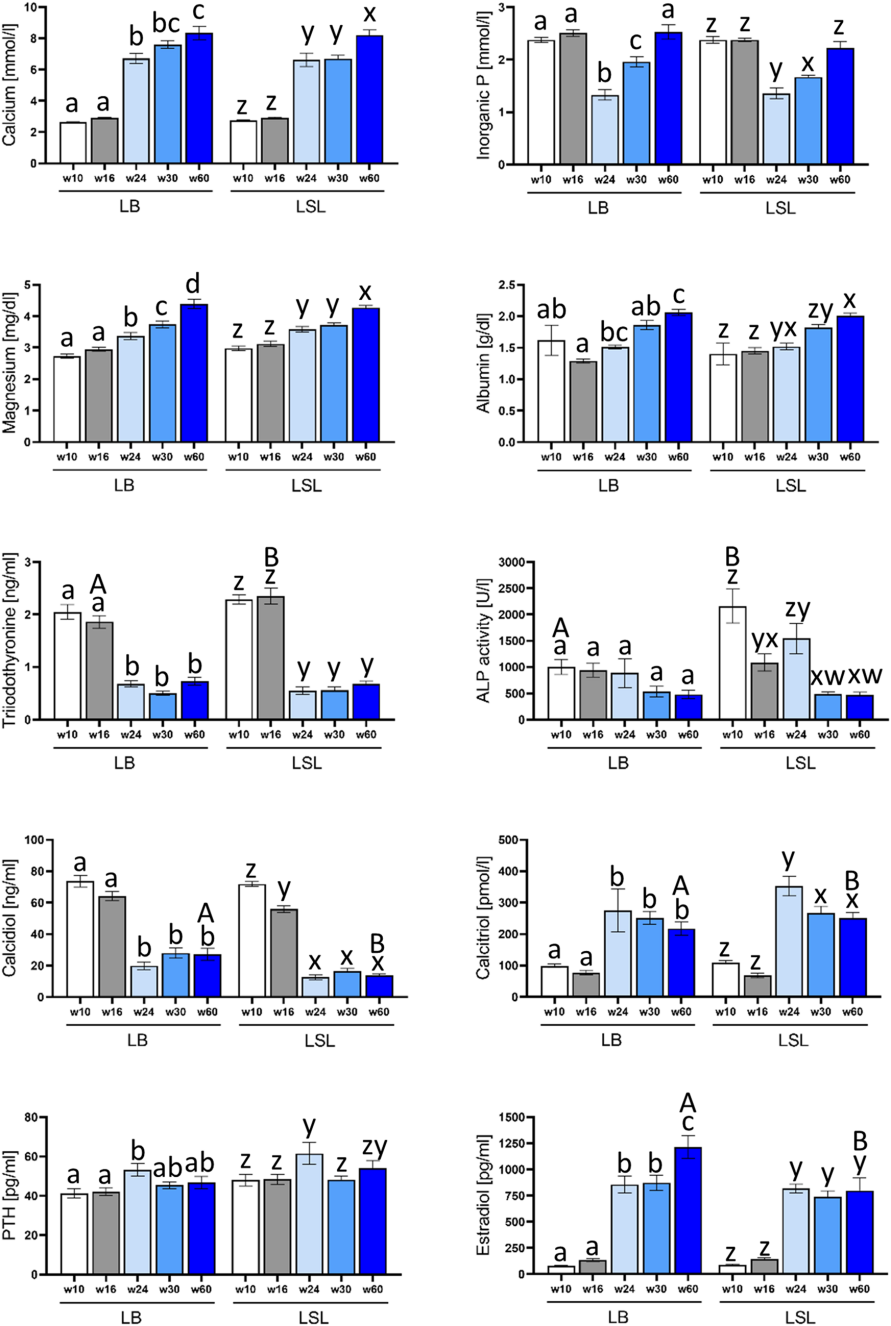
Plasma parameters referring to endogenous mechanisms to maintain mineral homeostasis with respect to the selected production stages (weeks 10, 16, 24, 30 and 60) of the LB and LSL laying hens. Values are displayed as means ± SE. Data for inorganic P and calcium were adopted from^9^. Superscripts indicate statistical significance (*P* < 0.05) between laying hen strains (capital letters) and production stages (small letters). PTH—parathyroid hormone; ALP—Alkaline phosphatase.

Plasma levels of calcium, magnesium and albumin showed production period-specific responses, which significantly increased along the developmental phases in both LB and LSL laying hens (Fig. 1, Table S1). The plasma levels of triiodothyronine (T3) and calcidiol (25OH-vitamin D3) were significantly lowered while levels of calcitriol (1,25 (OH)_2_-vitamin D3) and estradiol (E2) were significantly elevated from the onset of laying at week 24. A significant reduction with subsequent re-adjustment of the inorganic P levels was observed at the onset of laying at week 24 in both LB and LSL strains. The PTH levels were significantly increased at week 24 in both LB and LSL strains compared to other time periods. Alkaline phosphatase activity (ALP) significantly differed between growing and senescent LSL hens, while no significant differences between consecutive time points was observed in LB hens. Regarding strain differences, levels of triiodothyronine were found to be significantly higher in LSL strain compared to LB hens at week 16. For ALP, the activity was significantly higher at week 10 in the LSL as compared to LB hens. At week 60, estradiol and calcidiol levels were significantly higher in LB hens compared to LSL hens, while calcitriol was significantly higher in LSL hens compared to the LB hen strain. Notably, calcidiol levels at week 30 were numerically increased in LB compared to LSL strains (*p* = 0.051).

### Identification of differentially expressed genes (DEGs)

**Figure 2:**
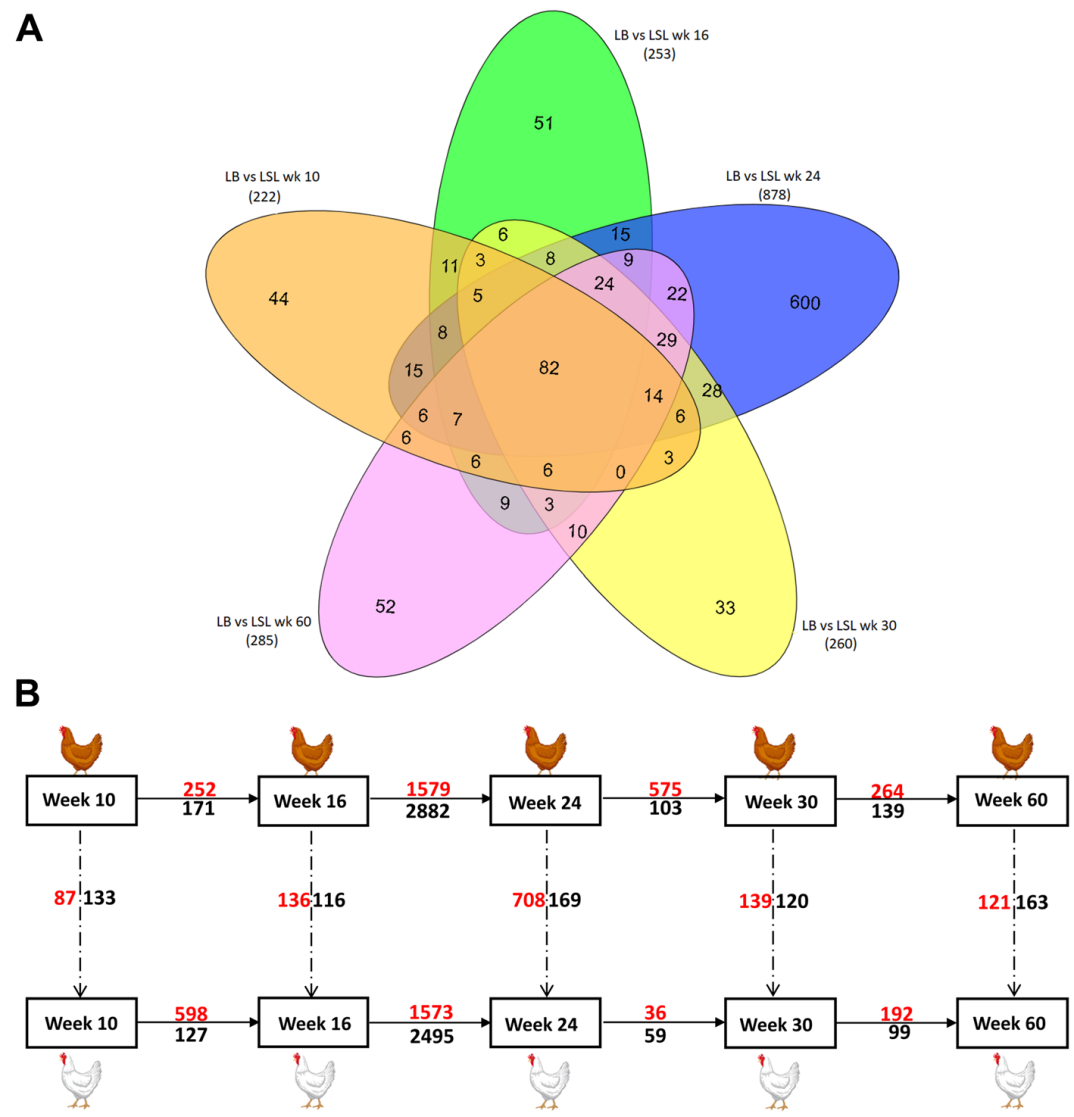
Number of DEGs derived from jejunal mucosa related to selected production stages in LB and LSL laying hens. (**A**) Venn diagram depicting the differentially expressed genes between LB and LSL for each production stage (week 10, 16, 24, 30 and 60) as well as their overlaps among different production stages. The total number of stage-specific DEGs is given in brackets. (**B**) Comparisons of DEGs within (horizontal) and between (vertical) each of the two laying hen strains across the selected production stages. Values in red and black represent numbers of upregulated and downregulated DEGs. LB—Lohmann Brown; LSL—Lohmann Selected Leghorn.

The DEGs were obtained by comparing the expression of jejunal mRNA from LB and LSL layer strains independently for each of the five production stages. The integration of the resultant DEGs revealed unique sets of production-stage specific genes found to be differentially abundant between both strains (Fig. 2A). In particular, 220 DEGs were identified between LB and LSL hens at week 10, while 262, 877, 259 and 284 DEGs were identified between the LB and LSL hens at weeks 16, 24, 30 and 60 respectively.

The strain comparison at pre-layer stages (week 10 and 16) revealed a total of 43 and 50 DEGs (Table S2). At the onset of laying in week 24, a total of 601 unique DEGs were identified, whereas weeks 30 and 60 showed 33 and 52 stage-specific DEGs, respectively, between LB and LSL strains. Interestingly, as the laying hens developed through the production periods, a total of 82 genes were consistently differentially expressed between both strains (Table S3). These specific differentially expressed genes over all the developmental stages were molecularly implicated in the immune modulation (*HCK*, *MTURN*, *CD8A*, *GBP6*), nucleotide-binding and chromosomal maintenance (*WRAP53*, *CELF5*, *MMRN2*), barrier integrity/extracellular matrix (*TMIGD1*, *COL9A1*, *LRFN5*, *CRTAC1*), and complex lipid synthesis (*SLC27A5*). Temporal DEGs exhibited within LB and LSL laying hen strains across the five production stages (Fig. 2B) were also deciphered by the comparison of the jejunal mRNA expression with the highest number of DEGs between week 16 and 24 (Table S4). The overlap of DEGs analyzed during this period is 69.5% (3399 genes) between the two strains.

### STEM, functional annotation and pathway enrichment analysis

**Figure 3:**
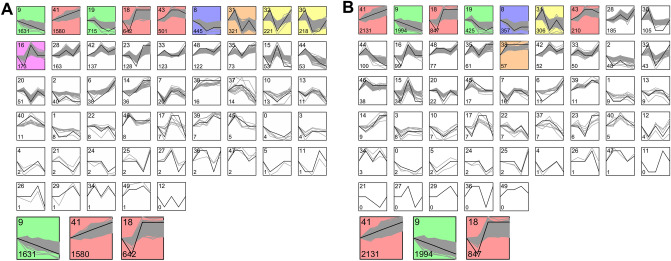
Time-series analysis of production stage-specific jejunal transcripts expressed in LB (**A**) and LSL (**B**). Expression patterns (grey lines) over the five selected production stages at week 10, 16, 24, 30 and 60 were clustered into profiles. Colors are assigned to only significant profiles (*p* < 0.05) and ordering is based on number of genes displayed in the lower left corner. The profile number is shown in the upper left corner. Highlighted profiles #9, #41, and #18 were selected for detailed analyses via IPA.

Considering the 5 production stages as time series, a total of 13,676 and 13,921 genes were used to analyze the transcriptional patterns in LB and LSL hens, respectively. The STEM analysis highlighted 10 significant profiles in the LB layers strain and 8 significant profiles in LSL (Fig. 3). Profiles #9 and #41 were selected for detailed analyses due to their linear time-course expression patterns in relation to the overall experiment. To approximate transcriptional shifts related to altered metabolic demands at onset of laying, profile #18 was considered for detailed analyses. Genes included in profile #18 represent a considerable overlap with DEGs identified in the contrast between week 16 and 24 (Supplementary Fig. S1). Moreover, additional DEGs from the week 16 to 24 comparisons are assigned to profiles #9 and #41.

**Figure 4:**
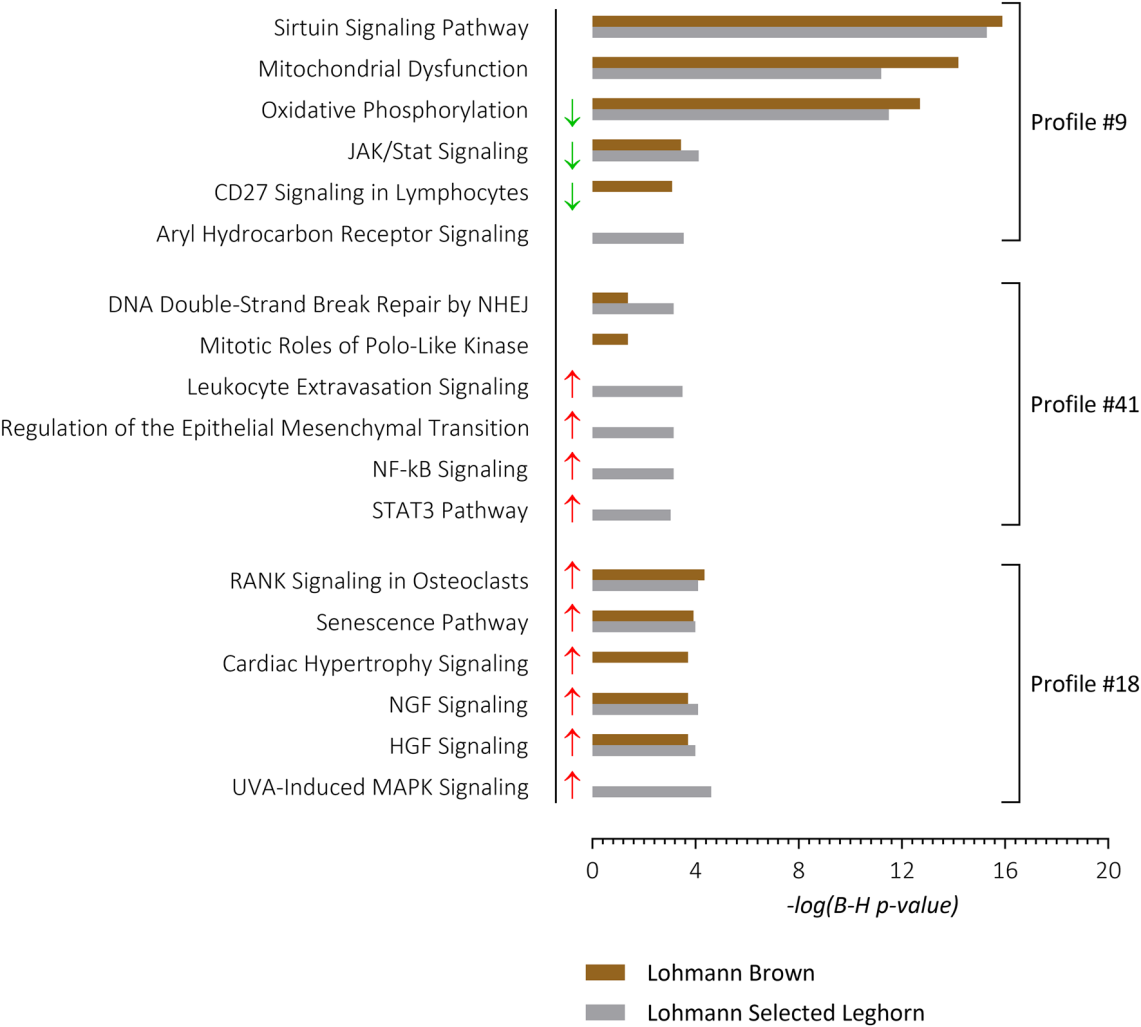
Top 5 canonical pathways predicted from selected STEM profiles in LB and LSL laying hens. Analyses comprise profiles #9 (consistently lower mRNA abundances with advancing production periods), #41 (consistently higher mRNA abundances with advancing production periods), and #18 (sustained increase in mRNA abundances from onset of laying). Arrows indicate significantly activated (red) and inhibited (green) pathways over the time course from week 10 to week 60 referring to the z-score. NHEJ—Non-homologous end joining.

Selected expression data from the STEM profiles were submitted to IPA for functional annotation analysis (Fig. 4, Tables S5, S6). Interestingly, genes clustered in profile #9 in the LB (1631 DEGs) and LSL (1994 DEGs) strains were involved in the mitochondrial energy transduction processes over the production stages, whereas profile #41 comprised genes enriched in the cell-cycle and mitosis/DNA damage regulation checkpoint prior to division and differentiation. Profile #18 exhibited enrichment in RANK/RANKL signaling and cellular senescence in LB and LSL layers.

## Discussion

The onset of egg production in the laying hen is preceded by a myriad of interconnected biological processes, which spans the endocrine secretion of hormones and feedback mechanisms among the target organ (ovary) and other organ systems (jejunum, bone, kidney, liver, parathyroid) to modulate nutrient and mineral utilization according to changing needs. The physiological shift into the onset of laying is characterized by an intensification of the calcium metabolism crucial for eggshell calcification^23^, which is driven exogenously by the adequate supply of dietary nutrients and endogenously by the osteoclastic resorption of the medullary bone, which serves as a calcium reservoir^14,24,25^. Furthermore, alongside calcium, mineral P is equally important owing to its numerous physiologic functions in skeletal development, blood buffering, mineral metabolism, and energy signaling^25,26^ which are pivotal for optimal production in the laying hen.

### Dynamics of mineral homeostasis throughout the production period

There was a considerable increase in plasma calcium levels and a reduction in plasma P levels with the onset of laying in both strains. This reflects the increase in dietary calcium at week 24 compared to weeks 10 and 16 which hampers intestinal phytate degradation and mineral digestibility resulting in lower P uptake^9^. However, PTH was increased at week 24 in both LB and LSL strains compared to pre-laying period. Indeed, PTH favors the endocrinal regulation of calcium homeostasis via activation of osteoclastic bone resorption and renal reabsorption of calcium^25,27^. Since the calcium content of the feed may not meet the immediate needs at the beginning of the laying period, an increase in PTH production triggers the mobilization of bone calcium reserves to match metabolic calcium requirements. However, intestinal capacity for mineral uptake is known to increase in laying hen at onset of laying^28^.

Consequently, at the laying peak (around week 30) a re-increase in plasma P was observed in both LB and LSL layers, which suggests adapted intestinal fluxes. Interestingly, calcitriol levels were increased from week 24 compared to the pre-laying period. In physiological conditions, increased calcitriol levels prompt decreased PTH levels^29,30^. However, in this study, PTH levels from week 30 onwards were still relatively high. This can be explained by the need to prevent a calcium deficiency due to a competing calcium demand for eggshell production and the associated fluctuation in calcium^23,31–33^. These regulations account for dramatic change in bone metabolism at sexual maturity driven by endocrine secretion. Consequently, triiodothyronine levels were decreasing while estradiol levels were found to be increased in both laying hen strains to induce egg laying capacity while terminating somatic growth and development^34–36^.

Estradiol, a most potent form of estrogen, is secreted principally by the ovaries of the hen and mediates the overall maturation and development of the female reproductive system. It has a regulatory role in the induction of vitellogenesis, the activation of yolk precursors in the liver^37^ and contributes to the formation of the medullary bone^15^. In this context, the vitamin D system undergoes dramatic changes, which implicates the regulation of mineral homeostasis via bone remodeling and resorption. At week 24, calcidiol levels dropped whereas calcitriol levels increased compared to the pre-laying period in both strains. It is conceivable that the synthesized calcidiol from liver is deposited in the egg yolk as embryonic reservoir^38^. However, the calcitriol level in conjunction with increased estradiol level account for osteoblastic formation of medullary bone during the entire productive period^39^ and thus provides a stock of mobilizable calcium. Notably, the consecutive increase on levels of plasma magnesium might counteract the very high calcium plasma levels and affect on blood viscosity^40^. The increasing albumin levels might account for egg production in both laying hen strains.

Regarding the observed strain effects, the levels of triiodothyronine were increased in LSL compared to LB laying hens at week 16. This might reflect compensatory response to the body growth since LSL hens have lower body weight compared to LB hens^9^. Furthermore, at week 60 the hen strains differed in plasma levels of estradiol (LB > LSL), calcidiol (LB > LSL), and calcitriol (LB < LSL). This reflects different strategies to ensure long-term metabolic demands. Beside the dietary shifts at the onset of laying, the endocrinal profiles clearly show a physiologic shift that leads to a pre-laying and an egg-laying period engaging a large number of organs including kidney, liver, bone, ovary, and jejunum.

### Longitudinal evaluation of jejunal gene expression throughout the production period

Transcriptomically, the 82 genes expressed differentially between the LB and LSL hens, consistently over all five developmental stages (Fig. 2A), are connected to biological processes along immunity (*CD8A*, *GBP6*, *HCK*), extra cellular matrix formation (*COL9A1*, *CRTAC1*, *MMRN2*), and micro- and macronutrient utilization (*HFE*, *SLC27A5*). Interestingly, the transcript abundance of avidin encoding gene (*AVD*) is consistently higher in LB compared to LSL irrespective of production stage, which suggests strain-specific alterations of biotin levels^41^. It is conceivable that these strain-specific transcript abundances are due to the observed genetic differences between LB and LSL laying hens that affect immune competence, e.g. resistance to endoparasite infection^42,43^. Additionally, the analyses of production stage-specific jejunal transcripts identified a number of genes within each strain that followed specific expression patterns (Fig. 3). The pathway analysis for the profiles #9, #41, and #18 for each strain over the developmental stages revealed enrichment in pathways, which spans mitochondrial energy transduction, cell-cycle regulation, DNA damage repair mechanisms and RANK/RANKL-induced immune modulation related to physiological growth and maturation (Fig. 4).

Genes assigned to profile #9 were gradually decreasing in expression throughout the production period. This profile highlighted overlapping pathways that encompass mitochondrial energy transduction and cellular growth processes in the LB and LSL layer strains, such as the sirtuin pathway, oxidative phosphorylation, mitochondria dysfunction, and JAK/STAT signaling. Members of the sirtuin family are nicotinamide dinucleotide (NAD+) dependent deacylases, of which *SIRT2*, *SIRT6* and *SIRT7* were enriched in profile #9. They are implicated in several molecular regulatory process e.g. cellular metabolism, energy metabolism, and cell survival. The sirtuin pathway enables effective adaptive response to metabolic, oxidative and genotoxic stress through metabolic homeostasis mechanism by acting as cellular sensors for energy abundance and modulating metabolic processes in conjunction with the mitochondria^44,45^.

Mitochondrial dysfunction and oxidative phosphorylation enriched in profile #9 corroborates the mitochondrial theory of ageing. Enriched genes represented all five complexes of the mitochondrial electron transport chain. This is indicative of an overall decline of energy-dependent processes (e.g. intestinal cell renewal and proliferation processes) which were considered optimal at an earlier stage of production, but possibly experienced a reactive oxidative species (ROS) associated decline over time^46,47^. ROS play essential roles in proper oxygen sensing, maintenance of cellular redox state, cell signaling and the regulation of cell proliferation and differentiation at lower concentrations^48–50^. However, the long-term accumulation of ROS with advancing age may result in the loss of the mitochondria integrity, functionality and ultimately dysfunction. Indeed, mitochondria are speculated to play a key role in delaying or accelerating the aging process especially in tissues with a high demand in energy^51^.

The JAK/STAT signaling pathway has been reported to modulate the adaptive and innate immune component of layers’ intestinal mucosal as well as epithelial repair and regeneration, via the activation of growth factors and cytokines^52^. In this regard, the transcription factor encoding genes *STAT2*, *STAT3*, *STAT5A* and *STAT6* were enriched in profile #9, indicating the transmission of effects at the level of gene expression represented by this pathway. The onset of laying in particular has been shown to have effects on the immune system as analyzed in the blood, spleen, and cecal tonsils of LB and LSL laying hens^12^. Moreover, corresponding analyses in the same individuals highlighted regulatory roles of miRNA within the JAK/STAT signaling^53^. The significant inhibition of the JAK/STAT signaling pathway and of oxidative phosphorylation over the developmental stages from week 10 to week 60 in both laying hen strains suggests a gradual shift in resource allocation from the initial modulation of cellular growth processes to the maintenance of the intestinal epithelium.

Genes allotted to profile #41 showed an increasing trend over the developmental stages whereby in both laying hen strains these mainly involve different molecular pathways, i.e. pathways related to cell cycle regulation and cell division in LB and pathways related to immunity and regulation of epithelial repair and regeneration in LSL. Specifically, pathways related to the innate immune system involving leukocytes and NF-κB, as the main regulator of innate immune responses, were shown to be activated in LSL with increasing age^54^. This buttresses the adaptive responses of the LSL strain via efficiency in paracellular transport and immune competence^10^. An overlap in predicted pathways of both hen strains was observed for DNA Double-Strand Break Repair by Non-Homologous End Joining, as evidenced by the clustered expression patterns of *ATM*, *DCLRE1C*, *LIG4*, *MRE11*, *PARP1* and *XRCC5*. This might reflect the accumulating number of senescent gut cells in both layer strains over the production stages and aging.

The expression profiles of genes assigned to profile #18 showed a considerable and sustained increase in expression with the beginning of the laying period in week 24. Most of the highlighted pathways based on this profile, including RANK signaling, senescence pathways, HGF and NGF signaling pathways, overlapped between the two laying hen strains. Due to the pattern, direct effects of dietary change or secondary effects of sexual maturity and the nutrient demand with the onset of lay are conceivable^55^. The direct dietary effects would be applicable to the enrichment of the cellular senescence pathways, which might occur due to the fourfold increase of calcium content in the diet and corresponding changes in gastrointestinal pH and microbiota.

Furthermore, RANK signaling has been associated with the gastrointestinal tract through its pro-immune activities within the epithelium, specifically, via the mediation of the development and differentiation of sentinel M cells present in the follicle-associated epithelium (FAE) which covers the gut-associated lymphoid tissues (GALT)^56–58^. In adaptation to the onset of egg laying, the endogenous release of calcium to meet production demand occurs under the collaborative actions of endocrinal pro-resorption factors such as calcitriol, PTH and estradiol, in conjunction with transcriptional modulation of the RANKL/RANK signaling pathway^59–62^.

The RANK signaling pathway was predicted to be activated in both laying hen strains with an increase in the expression over the time course from week 10 to week 60, i.e., a steady low expression of pro-bone resorption DEGs at the pre-lay stages, followed by a surge at the onset and peak of production, possibly due to the increased metabolic demands during production and, a plateau in the post-peak production stage, which is reflective of senescence. Additionally, the HGF and NGF signaling pathways were predicted to be activated in both hen strains, suggesting an increased gut-brain crosstalk for the attainment of enteric homeostasis over the production periods^62^.

## Materials and methods

### Ethical statement

The animal experimentation was performed at the Agricultural Experiment Station of the University of Hohenheim, Germany, in accordance with relevant guidelines and regulations and approved by the Animal Welfare Committee of the University of Hohenheim. The experimental protocol is in strict compliance with the German Animal Welfare Legislation and approved by the Regierungspräsidium Tübingen, Germany (Project No.: HOH50/17TE) and in accordance with the ARRIVE guidelines.

### Experimental chicken population and sample collection

**Figure 5:**
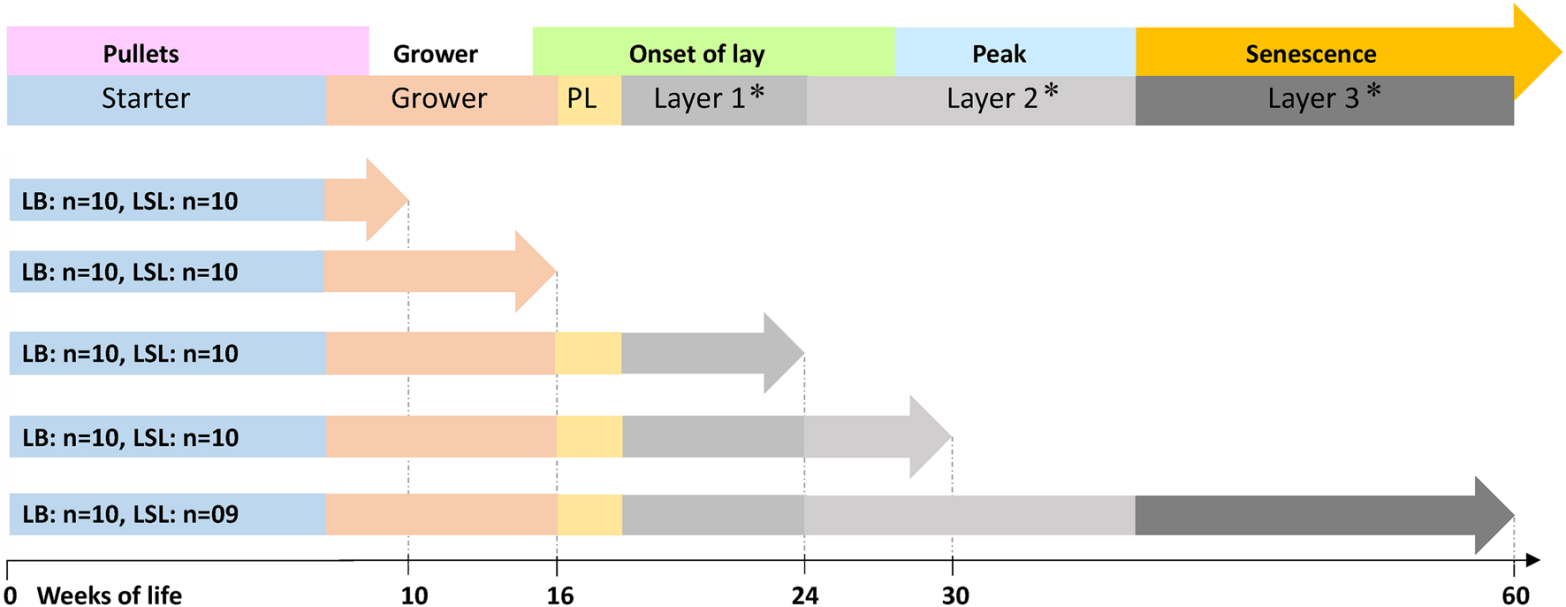
Dietary regimen for LB and LSL laying hen strains throughout the entire production period. Jejunal mucosa scrapings and plasma samples were retrieved at week 10, 16, 24, 30, and 60 to cover relevant production stages. Asterisks indicate a four-fold increase in the dietary calcium level to address the physiological demands from the onset of egg production. LB—Lohmann Brown; LSL—Lohmann Selected Leghorn; PL—pre-laying.

Two strains of laying hens were used for this trial (Fig. 5). As described previously, LB (n = 50) and LSL (n = 50) laying hens were fed a corn-soybean based diet with recommended calcium levels^9^. The feed formulations covered starter, grower, pre-laying (PL), and laying diets (layer 1, layer 2, layer 3)^9^. In all formulations, plant-based phytases were minimized and exogenous phytases of microbial origin were not included^9^. Birds were sampled at weeks 10, 16, 24, 30 and 60 of life to cover relevant periods of the production cycle, i.e. pullets, pre-layer, onset of laying, peak of laying, and senescence. The sampling comprised ten birds per strain with the progeny of the same ten fathers per strain at each of the sampling stages. Following stunning, hens were sacrificed by exsanguination at 0900–1200 h. Plasma samples were prepared from trunk blood in heparin-containing tubes by centrifugation (10 min at 2500×g) and stored at − 80 °C until analysis. After removal of the gastrointestinal tract, a 2 cm jejunum samples were collected approximately 3 cm distal to the duodenal loop. The samples were cut open, the mucosa was thoroughly rinsed with a 0.9% NaCl solution and scraped for each bird over the respective production stages (LB, n = 50; LSL, n = 49). Samples were frozen on dry ice and stored at − 80 °C until RNA extraction.

### Measurement of blood parameters

The levels of albumin, magnesium and alkaline phosphatase activity were analysed in plasma samples using the Fuji DriChem 4000i commercial assays (FujiFilm, Minato, Japan). The calcium and phosphorus values of the same samples were determined photometrically as part of the previous work^9^ Hormones were measured in duplicate using commercially available enzyme-linked immunosorbent assays (ELISA). ELISA kits were used with strict adherence to the manufacturer’s instructions for estradiol (EIA-2693, DRG, Marburg, Germany), 1,25(OH) vitamin D (AC-62F1, Immunodiagnostic Systems GmbH, Frankfurt am Main, Germany), triiodothyronine (EIA-4569, DRG, Marburg, Germany), parathyroid hormone (CSB-E118880Ch, CusaBio, Houston, USA) and 25(OH) vitamin D (EIA-5396, DRG, Marburg, Germany). For data analysis, a linear model was applied including the production stages, laying hen strains, hen father and slaughter order with the ‘lm’ function of the ‘stats’ package^63^. Pairwise comparison of means between experimental groups was achieved with the Tukey posthoc statistics embedded in ‘stats’ R package. Differences between hen strains and production stage were considered significant at *P* < 0.05.

### RNA extraction and sequencing

Total RNA was isolated with TRIzol Reagent (Invitrogen, Karlsruhe, Germany) from all 99 jejunal samples RNA was purified with the RNeasy Mini Spin kit including an additional DNase digestion (Qiagen, Hilden, Germany). The quantity and quality of final RNA were determined through spectrophotometry using the NanoDrop ND-2000 (Peqlab, Erlangen, Germany) and Bioanalyzer 2100 devices (Agilent Technologies, Waldbronn, Germany). RNA integrity numbers (RIN) were between 7.0 and 9.6. Sequencing libraries with a unique index for each sample were generated via stranded mRNA library preparation kit (Illumina, San Diego, CA, USA). Prior to sequencing, individual libraries were pooled. Paired-end sequencing was performed on a Illumina HiSeq 2500 device with 2 × 101 bp reads. Retrieved raw data were provided to the EMBL-EBI (www.ebi.ac.uk/arrayexpress) database (E-MTAB-9137).

### Processing and analysis of gene expression analysis

Quality control and preprocessing of raw sequencing reads was performed using FastQC (version 0.11.7) and Trim Galore (version 0.5.0; https://www.bioinformatics.babraham.ac.uk/projects/). Low-quality reads (mean Q-score < 20) and short length reads (< 30 bp) were removed. The resulting reads were mapped to the chicken genome assembly (GRCg6a, Ensembl release 95) using Hisat2 (version 2.1.0; http://daehwankimlab.github.io/hisat2/). Read counts for each gene were summarized with HTseq (version 0.11.2)^64^. The average number of pair-end reads per jejunal sample was 20.0 ± 2.9 million. The entire dataset was checked for sample outliers using the arrayQualityMetrics package in R^65^. Subsequently, differentially expressed genes (DEGs) were retrieved via DESeq2 applying the in-build normalization method^66^. The count data were initially filtered to remove very low abundant transcripts and retain observations with 5 or more counts in at least 8 animals of the entire data set. For comparison of the two laying hen strains within each production stage, a base model to identify DEGs was performed using the DESeq2^66^. In order to identify DEGs in the contrasts of the production stage within each of the two strains an additional a statistical model was applied including hen father as a fixed effect. DEGs met the criteria of p-value < 0.01 and |Log2FC|> 1.5. Q-values were estimated to calculate the false positive rate < 0.01^67^. Differentially expressed genes revealed in the contrast of the production stages between both layer strains LB and LSL were visualized using the InteractiVenn^68^.

### Gene clustering using short time-series expression miner (STEM)

STEM, a java application suitable for the analysis of longitudinal gene expression data^69^, was employed to gain insight into the temporal expression of genes via the comparison, clustering and visualization of expression patterns and their associated genes over the 5 production stages in the LB and LSL layer strains. Therefore, count-based data was transformed to regularized log values over all production stages for the two-layer strains. The median of individual values was generated per production stage and strain and submitted for the STEM analysis. The STEM clustering method was adopted with filtering threshold at a false discovery rate (FDR) < 0.05^70^.

### Functional annotation and pathway enrichment analysis of DEGs

Initially, the online tool g:profiler was used to convert the chicken Ensembl IDs to human orthologue gene symbols (https://biit.cs.ut.ee/gprofiler/orth)^71^. Ingenuity Pathway Analysis (IPA, Qiagen Redwood City, www.qiagen.com/ingenuity) was used to further derive biological interpretation of the resultant profiles from STEM. Temporal differentially expressed genes clustered per profile, along with their corresponding base-mean values, gene symbols and fold changes for the entire production period (week 10 vs. week 60) were submitted to IPA for the identification of canonical pathways based on the Ingenuity® Knowledge Base. Human orthologous gene symbols for 12,047 (LB) and 12,214 (LSL) chicken transcripts were considered in IPA analysis. Canonical pathway significance was tested at an adjusted P-value (Benjamini-Hochberg) < 0.05. Pathways were considered significantly activated or inactivated at an IPA-predicted absolute z-score > 2. Cancer-related pathways were excluded from the results derived from IPA.

## Conclusions

The onset of egg production, its peak, and senescence involve a cascade of several biological complexes, which are characterized by the interrelatedness of diet and physiological transition mediated by endocrinal regulation and transcript expression at each production stage. The attainment of sexual maturity in laying hens and its associated shift in dietary calcium intake at onset of egg production proves to be the most crucial developmental stage in the entire production cycle as proven by the conspicuous shifts in blood plasma metabolites levels. In particular, the high calcium requirement from the start of the laying required subtle coordination between PTH and the vitamin D system from week 24, which seems crucial to ameliorate production. Thus, the transcriptomic investigation of the jejunum from LB and LSL laying hens revealed several signaling pathways substantiating the complexity and importance of the jejunum in its contribution to the overall health and maintenance for optimum production across the entire developmental period in the layers. The study shows that both strains cope with changes in metabolic demands to reach comparable egg production performance by partially recruiting different pathways. The strains differ in pathways related to immunity, barrier and age-related tissue and cell integrity during all production periods, which could be due to genetic differences between the strains and deserve further investigation. However, insights into the host-microbiota interaction, specifically its influence on the gut-brain complex will further strengthen the knowledge and facilitate the management to improve mineral utilization and egg production.

## Supplementary Information

Supplementary Information 1.

Supplementary Information 2.

Supplementary Information 3.

Supplementary Information 4.

Supplementary Information 5.

Supplementary Information 6.

Supplementary Information 7.

## Data Availability

The raw data were deposited in the EMBL-EBI (www.ebi.ac.uk/arrayexpress) database under accession number E-MTAB-9137.
