# Supplementary material for: Jejunal transcriptomic profiling of two layer strains throughout the entire production period
Source: Sci Rep. 2021 Oct 11;11:20086. doi: 10.1038/s41598-021-99566-5 (PMC8505660; doi:10.1038/s41598-021-99566-5)

**Supplementary Figure S1.** Venn diagram combining gene lists retrieved from the time series expression analyses (profiles #9, #18, #41) and differential gene expression between weeks 16 and 24 in LB (A) and LSL laying hens (B).


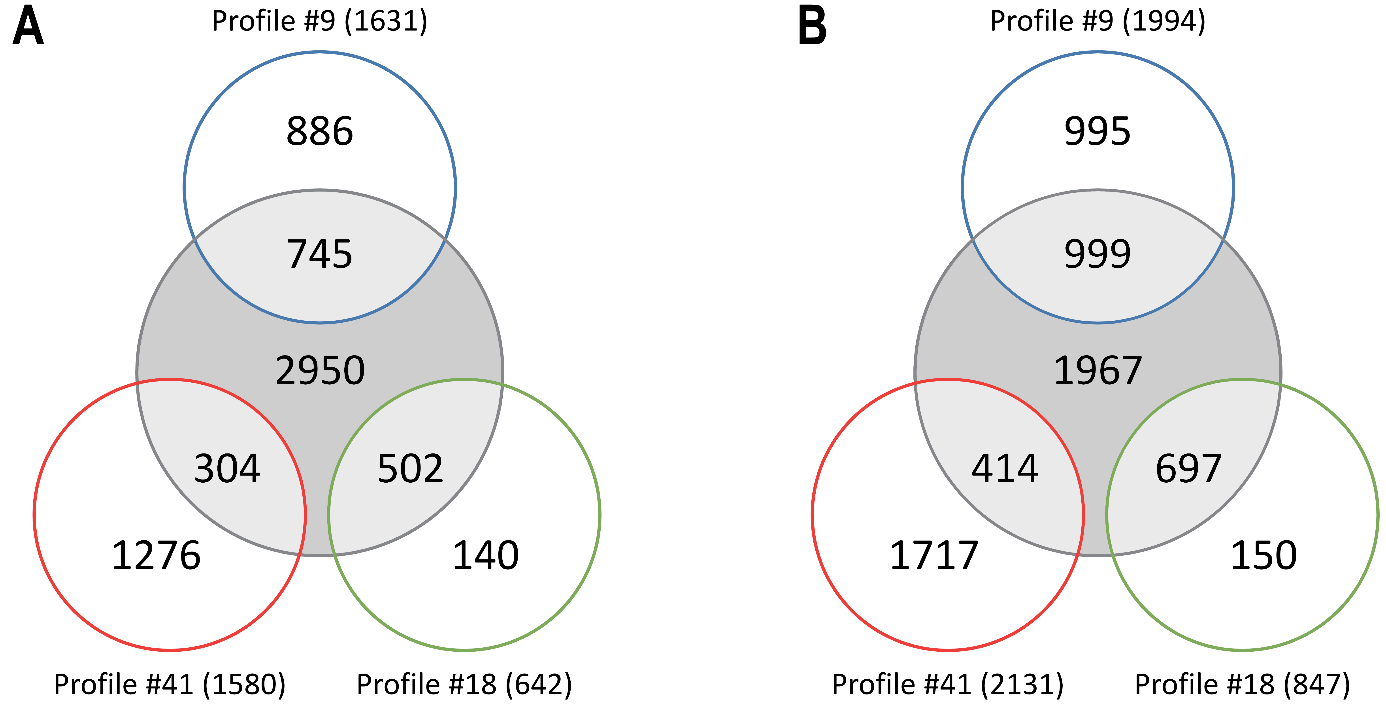

Supplement: Supplementary file 1 — Supplementary Information 1. [file 41598_2021_99566_MOESM1_ESM.docx]
